# Safety and Efficacy of amplitude-modulated radiofrequency electromagnetic fields in advanced hepatocellular carcinoma

**DOI:** 10.1051/fopen/2021003

**Published:** 2021-07-21

**Authors:** Arthur W. Blackstock, Al B. Benson, Masatoshi Kudo, Hugo Jimenez, Preeya F. Achari, Callum McGrath, Volker Kirchner, Lynne I. Wagner, Nathaniel S. O’Connell, Kathy Walker, Valerie K. Pasche, Ralph D’Agostino, Alexandre Barbault, Boris Pasche

**Affiliations:** 1 Department of Radiation Oncology, Wake Forest Baptist Medical Center, Winston-Salem, NC 27157, USA; 2 Wake Forest Baptist Comprehensive Cancer Center, Winston-Salem, NC 27157, USA; 3 Division of Hematology/Oncology, Northwestern Medical Group, Chicago, IL 60611, USA; 4 Department of Gastroenterology and Hepatology, Kindai University Faculty of Medicine, Osaka 577-8502, Japan; 5 Department of Cancer Biology, Wake Forest Baptist Medical Center, Winston-Salem, NC 27157, USA; 6 Genolier Cancer Center, Genolier 1272, Switzerland; 7 Department of Social Sciences and Health Policy, Wake Forest University Health Service, Winston-Salem, NC 27157, USA; 8 Department of Biostatistics and Data Science, Wake Forest Baptist Medical Center, Winston-Salem, NC 27157, USA; 9 TheraBionic Inc., Winston-Salem, NC 27106, USA; 10 TheraBionic GmbH, Ettlingen 76275, Germany

**Keywords:** TheraBionic, Hepatocellular Carcinoma, Amplitude-Modulated, Radio Frequency, Electromagnetic Fields, Sorafenib, AM RF EMF

## Abstract

**Importance:**

Hepatocellular carcinoma (HCC) is the third leading cause of cancer death worldwide. Despite the recent approval of several new agents, long-term disease control remains elusive for most patients. Administration of 27.12 MHz radiofrequency (RF) electromagnetic fields (EMF) by means of a spoon-shaped antenna (TheraBionic P1 device) placed on the anterior part of the tongue results in systemic delivery of low and safe levels of RF EMF from head to toe.

**Objective:**

To report treatment outcomes and adverse events associated with treatment with the TheraBionic P1 device in comparison to suitable historical placebo and actively treated controls.

**Design:**

Pooled case series with comparison to historical controls.

**Participants:**

Patients with advanced HCC receiving this treatment, 18 real-world patients and 41 patients from a previously reported phase II study. Historical controls from previously conducted clinical trials.

**Interventions:**

Three hours daily treatment with the TheraBionic P1 device compared with standard of care as received by historical controls in the previously conducted trials.

**Main outcomes and measures:**

Overall survival (OS), time to progression, response rate, and adverse events in the combined pooled patients and in appropriate subgroups comparable to the historical control groups.

**Results:**

In the pooled treatment group, median OS of patients with Child-Pugh A disease (*n* = 32) was 10.36 (95% CI 5.42–14.07) months, 4.44 (95% CI 1.64–7.13) months for patients with Child-Pugh B disease (*n* = 25), and 1.99 (95% CI 0.76–3.22) months for patients with Child-Pugh C disease (*n* = 2). Median OS for Child-Pugh A patients was 2.62 (33.9%) months longer than the 7.74 months OS of comparable historical controls (*p* = 0.036). The 4.73 (95% CI 1.18–8.28) months median OS for Child-Pugh B patients receiving TheraBionic P1 device as first line therapy is slightly higher than the 4.6 months median OS of historical controls receiving Sorafenib as first line therapy. Only grade 1 mucositis and fatigue were reported by patients using the device, even among Child-Pugh B and C patients. No patients discontinued treatment because of adverse events.

**Conclusions and Relevance:**

Treatment of advanced HCC with the TheraBionic P1 device is well tolerated, even in patients with severely impaired liver function, and results in improved overall survival compared to historical controls without any significant adverse events, even after many years of continuous treatment. This treatment modality appears to be well suited for patients who have failed or are intolerant to currently approved therapies.

## Introduction

Primary liver cancer is the second leading cause of cancer death worldwide. In 2020, there were 905,677 new cases and 830,180 deaths from liver cancer in 2020 [1]. Hepatocellular carcinoma (HCC) is the most prevalent type of liver cancer, which accounts for approximately 75% of all liver cancers globally [2]. Although the field of advanced HCC care has changed rapidly over the past few years with the approval of several new drug treatments [3–6], none of these treatments are curative. Virtually all patients with advanced HCC will eventually become untreatable, the majority because of impaired liver function, which precludes the use of most currently available therapies. Indeed, only one drug (Nivolumab) is currently recommended for patients with severely impaired liver function, i.e., Child Pugh B8 or B9 (NCCN Hepatobiliary Guidelines, version 1.2021, March 2021), and real-world data shows that the median survival of patients receiving Nivolumab after Sorafenib failure is only 7 weeks [7]. Hence, HCC prognosis remains poor and long-term control of the disease is elusive for most patients.

Systemic treatment of cancer with low and safe levels of 27.12 MHz radiofrequency electromagnetic fields is a novel treatment approach, which has shown anticancer activity in several tumor types, including advanced HCC [8, 9]. *In vitro* and *in vivo* experiments have demonstrated that sequential amplitude modulation of a 27.12 MHz carrier frequency at tumor-specific frequencies identified in patients and ranging from 0.1 Hz to 150 kHz [8] blocks the growth of HCC tumors and results in the differentiation of tumor cells into scar tissue [10, 11]. HCC-specific frequencies exert antiproliferative effects in both hepatitis B positive and hepatitis B negative HCC cells and activate Cav3.2 T-type Voltage-Gated Calcium Channels (VGCC) leading to transient calcium influx and activation of the inositol-1,4,5-trisphosphate – diacylglycerol (IP3/DAG) signaling pathway [10]. A battery-powered portable radiofrequency emitting device connected to a coaxial cable ending with a spoon-shaped antenna placed on the anterior part of the patient’s tongue delivers HCC-specific frequencies to the patient’s entire body. This results in a whole-body Specific Absorption Rate (SAR) of 0.2–1 mW/kg with 1 g peak spatial SAR of 146–352 mW/kg [10]. Treatment is administered three times a day for one hour. This approach has been developed for the targeted systemic treatment of advanced hepatocellular carcinoma because it exerts its anticancer activity throughout the body and does not affect non-tumor cells [10, 11].

The TheraBionic device received European regulatory approval in 2018 as a class IIa low risk medical device according to the Medical Device Directives (MDD) 92/42/EEC guidelines and ISO 1345:2016 quality management systems regulatory requirements for medical devices [12]. Class IIa devices are defined as low to medium risk and include hearing aids and surgical gloves and the Thera Bionic device was approved in consideration of MDD 92/42/EEC MEDical DEVice Documents (MEDDEV) 2.7/1 rev. 4 Annex 8 regarding devices for unmet medical needs. The TheraBionic device received Breakthrough Designation from the FDA in 2019.

Here, we report real-world data from 18 patients with advanced HCC who received treatment with the Thera Bionic P1 device. We also report a combined analysis of the previously published data with real-world data as it relates to overall survival, progression-free survival, response rate and patient-rated symptoms.

## Methods

This study was approved by the Wake Forest Baptist Comprehensive Cancer Center Investigational Review Board (IRB # 00074844). All patients who received treatment with the TheraBionic device programmed with HCC specific frequencies and for whom TheraBionic Device Registry Forms (TDRFs) were available were included in this analysis. The phase I/II study included 41 patients from a single center [9]. The 18 patients described in this report were from multiple centers. Exclusion criteria for the phase I/II study were brain metastases, Child-Pugh C liver function, history of liver transplantation, stable disease or response after prior treatment, and pregnancy. Exclusion criteria for the compassionate use patients were the same with the exception that patients with Child-Pugh C disease were allowed.

Survival data and patient-rated symptoms according to National Cancer Institute (NCI) Common Terminology Criteria for Adverse Events (CTCAE) were extracted from the TheraBionic phase I/II study [9] Case Report Forms (CRFs) and from the TDRFs for patients who received treatment with the TheraBionic device in the feasibility study [8], on compassionate use or post CE approval. The TDRFs capture the same information as that captured by the phase I/II study, including demographics, prior treatment history, liver function laboratory results, ascites, encephalopathy, target lesions per RECIST 1.1 and mRECIST criteria, performance status, history of hepatitis, symptoms prior to treatment, symptoms while receiving treatment using NCI CTCAE criteria, and adverse device events. Patient-rated symptoms were assessed prior to TheraBionic treatment initiation and at each follow up visit during treatment using the NCI CTCAE version 3 for the phase I/II study and version 5 for the real-world patients. Return visits were scheduled every two months, more often when clinically indicated. Response assessment was conducted using the RECIST criteria [13] for the phase I/II study, and the revised RECIST criteria (version 1.1) [14] for the real-world patients. The best overall response had to be confirmed within two months.

Survival analysis methods were used to compare survival rates among Child-Pugh A, B, and C patients. Kaplan–Meier curves were generated and comparisons were made using the log-rank test. In addition, median survival times were estimated with 95% confidence intervals for different groups of interest using survival analysis models. Median time to event (i.e., death or progression) data was compared to historic median values using Wilcoxon signed rank tests. All analyses were performed using SAS version 9.4. Virtually all randomized studies in advanced HCC in the past decade only included patients with Child-Pugh A disease. Consequently, reliable overall survival data from well-controlled randomized studies that include a placebo arm is only available for this subgroup of patients. We decided, therefore, to compare the overall survival of patients with Child-Pugh A disease treated with the TheraBionic device (*n* = 32) with the overall survival of the placebo group from all randomized studies with a placebo arm published until 2019, which include two studies investigating first line therapy and nine studies investigating second line therapy [15]. Inclusion of both first line and second line studies is justified by the fact that 13 (40.6%) of the 32 Child-Pugh A patients treated with the TheraBionic device had received at least one line of systemic therapy prior to treatment initiation with the device. Three (9.4%) patients had received at least two different systemic therapies prior to treatment initiation with the TheraBionic device. The time period during which these studies were conducted also overlaps with the conduct of the phase I/II study and treatment of patients on compassionate use. The pooled overall median of the placebo arm of these studies was compared to the TheraBionic median using a Wilcoxon signed rank test.

In order to explore comparisons of TheraBionic to other active treatments, we used the following exploratory approach which generates a measure similar to the confidence interval of the difference between treatments.

We tested the observed median values from the TheraBionic data using the Wilcoxon signed rank against different potential median values of active comparator. Once we detected the first median level that was statistically significantly lower than the observed TheraBionic median using this statistical test we then compared that median level to the observed, pooled median value that was available from the published literature. The difference between these two values was considered the confidence interval of the treatment difference, so for example, if the TheraBionic observed median was 10.4 months and the published literature median was 11.1 months, and the first value that could be rejected was 8.1 months then the confidence interval of the treatment difference would be 3 months (11.1–8.1). This would suggest that the TheraBionic median value could be as much as 3 months lower than the published median, but not more than that amount.

We summarized patient adverse event (AE) data across the current study (*n* = 18) and the previously conducted Phase I/II study (*n* = 42). For each study, both separately and combined, we summarize the incidence of each AE grade individually, any grade AE, and grades 3 and 4 AE. For each summary table, we present the incidence of patient experienced AEs by maximum grade post-baseline and baseline adjusted approach proposed by Basch et al. [16]. Following the latter approach, baseline adjusted AEs are calculated for each AE such that if a patient’s maximum grade AE post-baseline is less than or equal to their baseline AE, their adjusted AE grade is 0. Otherwise, if a patient’s max-grade AE is greater than their baseline AE, their adjusted AE grade is equal to their max-grade AE. Additionally, we compare the AEs experienced by patients in the Phase I/II study (*n* = 42) to AEs reported in two Sorafenib trials [2, 3]. Specifically, for each overlapping AE experienced in both the TheraBionic phase I/II trial and the Sorafenib trials, we compare statistically using a Fisher’s Exact Test.

Primary data was reviewed independently by the following authors, H.J., P.A., C.M., V.K.P. and B.P., and tabulated in an excel spreadsheet. Data analysis examining time to event data (i.e., survival or progression) was conducted by one author (R.D. Jr.) using SAS ProcSAS version 9.4 Lifetest Procedure.

## Results

### Real-world data

Table 1 shows the demographics of the 18 real-world patients, which include all patients from the TheraBionic registry with a diagnosis of advanced HCC who received treatment with HCC-specific frequencies. The median age was 71. Fifteen (83.3%) patients were male. Eastern Cooperative Oncology Group (ECOG) performance status ranged from 1 to 3. Twelve patients had Child-Pugh A, four patients Child-Pugh B, and two patients had Child-Pugh C liver function. Half of the patients had serum Alpha-Fetoprotein (AFP) levels greater than 400 ng/mL. Fifteen (83.3%) patients had evidence of disease progression and all patients except for one had received at least one systemic therapy prior to initiation of treatment with the Thera Bionic device. Table 2 summarizes the treatments received prior to treatment with the TheraBionic device. Sorafenib was the most used anticancer therapy by 14 (77.8%) patients. In addition to 17 patients who had received 1 systemic therapy, three had received 2 systemic therapies, one 4 systemic therapies, and one 6 systemic therapies prior to treatment initiation with the TheraBionic device. The clinical data of the 41 patients from the phase I/II are published [9].

With a cutoff date of June 7, 2021, one patient is alive and receiving single modality treatment with the Thera Bionic device. The other patients have expired. The median overall survival of these patients is 6.67 (95% CI 4.44–9.50) months, which is identical to the 6.7 (95% CI 3.0–10.2) months median overall survival (OS) of patients enrolled in previously published phase I/II study [9]. Two (11.1%) of the 18 patients had a partial response (PR) as assessed by RECIST criteria, a PR rate similar to the 9.8% observed in the phase I/II study.

#### Overall survival

**Figure 1.**
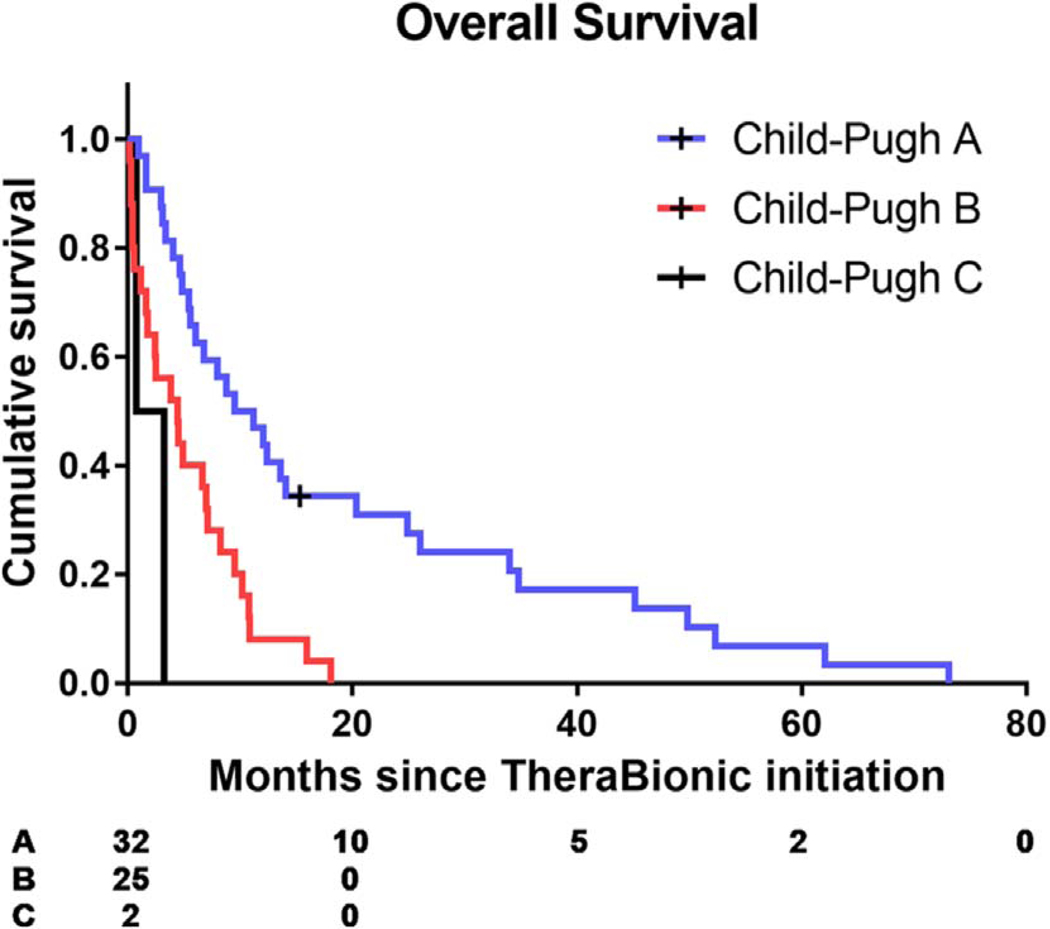
Survival curves of patients receiving treatment with the TheraBionic device. OS of 59 patients treated with the TheraBionic device stratified by Child-Pugh status (A/B/C).

Fifty-nine patients receiving TheraBionic treatment were included in these analyses. The median overall survival was 6.72 (95% CI 4.53–9.53) months. The median overall survival in the Child-Pugh A group (*n* = 32) was 10.36 (95% CI 5.42–14.07) months. The median overall survival in the Child-Pugh B group (*n* = 25) was 4.44 (95% CI 1.64–7.13) months. The median OS in the Child-Pugh C group (*n* = 2) was 1.99 (95% CI 0.76–3.22) months. Overall survival was compared among these three groups (Child-Pugh A, B and C) using a log-rank test and was found to be significantly different (*p* = 0.0007). The Log-Rank test (*p* = 0.0007), Wilcoxon Test (*p* = 0.0027) and the −2 Log (likelihood ratio) test (*p* < 0.0001) performed using Proc Lifetest in SAS all indicated that there was a statistically significant difference in survival distributions among the three groups (Figure 1 – Survival Curves comparing OS (in months) among Child-Pugh A, B and C patients) lifetest procedure OS months).

For this test, the observed median survival for the TheraBionic group was 10.36 months. This estimate was based on using an overall survival time of 15.3 months for the patient who was alive on June 7, 2021. Using this approach, we compared the median (10.36) to a value of 7.74 based on calculating a weighted average (weights based on sample size) of medians presented in the meta-analysis. Using this approach, the Signed rank statistic (from the Wilcoxon signed rank test) was equal to 116 (*p* = 0.028). We repeated this test comparing the median (10.36) to a value of 7.61 months based on pooling the medians from the meta-analysis as a simple average of medians (i.e., weighting each study equally independent of sample size). For this comparison, the signed rank test statistic was 120 (*p* = 0.022). In summary, the median OS of patients with advanced HCC and Child-Pugh A liver function who receive treatment with the TheraBionic device is 33.9% longer than the median OS of the placebo arm of these 11 randomized studies (7.74 months). Importantly, several patients who received treatment with the TheraBionic device had received more than one line of systemic therapy prior to treatment with the TheraBionic device. This analysis was repeated after removing the REACH-2 study [4], a study that included only patients with a baseline Alpha-Fetoprotein (AFP) level of at least 400 ng/mL, a subgroup of patients with worse prognosis. The median OS of Child-Pugh A patients receiving TheraBionic was 2.6 (33.5%) months longer than the 7.76 months weighted average of medians from the ten studies (*p* = 0.0306).

To determine whether the survival benefit was potentially confounded by additional anticancer therapies used concomitantly with the TheraBionic device by 5 (15.6%) of the 32 Child-Pugh A patients, we conducted two additional analyses. First, we compared the median OS of Child-Pugh A patients enrolled in the TheraBionic phase I/II study with the placebo arm of the SHARP [17] and Asian-Pacific [18] Sorafenib studies. The latter two studies were conducted during the same time as the TheraBionic phase I/II study. Specifically, patient recruitment for the SHARP study, the Asian-Pacific Sorafenib study, and the TheraBionic phase I/II study occurred between March 2005 and April 2006, September 2005 and January 2007, and October 2005 to July 2007, respectively. Hence, enrollment for all three studies occurred prior to regulatory approval of Sorafenib. This also means that most patients from either the placebo arm of the SHARP and Asian-Pacific Sorafenib studies or the TheraBionic phase I/II study did not have access to any therapy with a known impact on overall survival. The 11.8 months median OS of Child-Pugh A patients from the TheraBionic phase I/II study was 4.64 (64.8%) months longer than the 7.16 months OS from the pooled estimated median of the SHARP (*n* = 602) and Asian-Pacific studies (*n* = 271). The difference was statistically significant (*p* = 0.0375). Second, we compared the median OS of all Child-Pugh A patients who received single modality TheraBionic treatment until death or censoring with the median OS of all Child-Pugh A placebo patients enrolled in the eleven studies described above. The median OS of the Child-Pugh A patients receiving single modality TheraBionic was 11.21 (95% CI 4.6–24.9) months, i.e., 3.47 (44.8%) months longer than the 7.74 months pooled estimated medians of the 11 studies placebo arms (*p* = 0.0438).

When we performed an exploratory analysis to determine the lower confidence interval limit of the treatment difference between TheraBionic and the treatment arms of the four randomized, placebo-controlled studies that led to the approval of Sorafenib, Regorafenib and Cabozantinib, i.e., the SHARP [17] and Asian-Pacific (AP) [18] Sorafenib studies, the Regorafenib RESORCE [19] study, and the Cabozantinib CELESTIAL [3] study, we determined this margin was 1.9 months. The observed median OS for the TheraBionic and SHARP/AP/RESORCE/CELESTIAL studies were 10.36 and 10.0 months respectively. In other words, based on this data, we can reject a hypothesis that the median OS in the TheraBionic patients is 8.1 months (or lower) in favor of the alternative that the median OS is above 8.1 months (*p* < 0.05), suggesting that the TheraBionic median would be no more than 1.9 months (10.0–8.1 = 1.9 months) lower than the observed pooled median OS in the active arms of the SHARP/AP/ RESORCE/CELESTIAL studies. It should be noted that the observed OS in the TheraBionic group (10.36 months) is higher than the pooled median OS estimate from the SHARP/AP/RESORCE/CELESTIAL studies (10.0 months), so as more data is collected, we anticipate that the confidence interval of the treatment difference will become smaller.

Having identified a survival benefit in Child-Pugh A patients, which was comparable to three approved tyrosine kinase inhibitors (TKIs), we sought to evaluate the impact of TheraBionic treatment on patients with Child Pugh B disease. We compared the observed median OS from the Child-Pugh B first line patients in the TheraBionic group (*n* = 20) to published values of median survival in a meta-analysis of Sorafenib as first-line therapy in patients with advanced Child-Pugh B HCC [20]. This meta-analysis includes the Child-Pugh B subset analysis [21] of the phase II study of Sorafenib in patients with Child-Pugh A and B in 137 patients [22]. These comparisons used a Wilcoxon signed rank test. For this test, the observed median OS for the TheraBionic group was 4.73 months and the estimated median OS of the meta-analysis was 4.6 months. The Signed rank statistic was equal to 17 (*p* = 0.55). This test suggests that the median OS in the TheraBionic group is comparable to that observed among Child-Pugh B patients receiving Sorafenib. The observed median in the TheraBionic group is higher than the Sorafenib group and the Signed Rank test suggests that the two groups are very comparable. A Sign test comparing the Thera Bionic data to the median value of 4.6 had a test statistic of 0 (*p* = 1.0), suggesting that exactly half of the Thera Bionic patients had a survival time above 4.6 months and half had a survival time below 4.6 months. The interquartile range of survival times in the TheraBionic group was 1.41 months to 8.91 months. In summary, the median OS of patients with advanced HCC and Child-Pugh B disease who received treatment with the TheraBionic device (4.73 months) as first line therapy was comparable to the median OS of patients with advanced HCC and Child-Pugh B liver function who received Sorafenib as first line therapy. There are currently no approved systemic therapies for Child-Pugh C patients, which renders any statistical analysis challenging. Two patients with Child-Pugh C10 and C11, respectively, received treatment with the TheraBionic device. Importantly, none of them developed any treatment related adverse events.

### Time to progression

Time to radiological progression (TTP) for the 20 Child-Pugh A patients from the TheraBionic phase I/II study was 3.9 (95% CI 1.8–5.3) months. This was 1.38 (54%) month longer than the pooled estimated medians (accounting for study sample size) from the SHARP and Asian-Pacific placebo TTP (2.52 months) (*p* = 0.0153). TTP for the 12 Child-Pugh A real-world patients was 4.35 (95% CI 3.40–18.95) months. The TTP for all Child-Pugh A TheraBionic patients was 4.25 (95% CI 3.10–5.30) months, which was 1.93 (90.6%) months longer than the 2.32 (95% CI 1.4–3.0) months TTP of nine of the 11 randomized placebo studies for which TTP was available [15].

### Response rate

Six (10.2%) of the 59 patients receiving treatment with the TheraBionic device had a partial response according to RECIST criteria, four (9.8%) in the phase I/II study and two (11.1%) real-world patients. Five of these patients had Child-Pugh A (4 A5, 1 A6) and one Child-Pugh B9 disease. Five of them had radiological evidence of disease progression and four of them had received systemic therapy prior to receiving treatment with the TheraBionic device. Three patients were hepatitis C positive. Two patients had AFP equal or greater than 400 ng/mL. The median OS of these 6 patients was 43.5 (95% CI 10.8–73.1) months. When the analysis was restricted to Child-Pugh A patients, median OS was 52.3 (13.6–73.1). Only one of these six patients received additional systemic therapy after beginning treatment with the TheraBionic device.

### Adverse events

Tables 3–8 present summary data for each grade AE, any grade AE, and grade 3 or 4 AEs as described above for the combined data (Real-world + phase I/II), real-world patients only, Phase I/II patients study only respectively, and Child-Pugh A patients only. AEs were compared with those of the SHARP and Asian-Pacific Sorafenib studies, which used the same AE reporting tool.

Table 3 enumerates AEs reported by the 59 patients receiving treatment with the TheraBionic device. Table 4 describes AEs reported by the 18 real-world patients receiving treatment with the TheraBionic device. Table 5 lists AEs reported during the conduct of the TheraBionic phase I/II study. Table 6 presents AEs reported by Child-Pugh A patients receiving treatment with the TheraBionic device. Table 7 presents a comparison of any-grade incidence rates of Abdominal Pain, Anorexia, Diarrhea, Fatigue, Hand-Foot Syndrome, and Nausea in comparison between that observed among patients receiving treatment with the TheraBionic device and each Sorafenib Trial. Fisher’s Exact tests were used for the statistical comparison.

Comparing the incidence of Sorafenib induced AEs to those AEs experienced in the TheraBionic Phase I/II trial, we find the incidence rates of any grade Diarrhea and Hand-Foot syndrome are significantly lower in the TheraBionic trial patients than in either Sorafenib trial (*p* < 0.01 for all comparisons). Also, none of the patients treated with the TheraBionic device reported alopecia. We find a significantly lower incidence rate of Anorexia in TheraBionic patients (1 patient; 2%) relative to the SHARP study (incidence rate of 14%) (*p* = 0.041), and a marginally significant difference compared to the Asia-Pacific study (incidence rate of 13%) (*p* = 0.081). Further, we do not find a statistically significant difference between trials in terms of incidence of any grade abdominal pain, fatigue, or nausea, although the incidence rate differences for Nausea were marginally significant (*p* = 0.10 and 0.129), with the rate in the TheraBionic being 2% (1 patient), compared to the 11% nausea rate observed in each Sorafenib trial. However, with specific regards to fatigue and abdominal pain, when assessing baseline adjusted AEs in the Phase I/II trial patients (Tab. 3), we find no instances of abdominal pain and only 1 instance of fatigue, suggesting the presence of these AEs at their maximum grade were already existent at baseline. While not statistically compared to the Sorafenib trials because Sorafenib trial baseline adjusted AE data is not available, in our current data (*n* = 18) summarized by Table 4, we find that only 4 baseline adjusted AEs manifested − 1 case of grade 1 Anorexia, 1 case of grade 1 GI Bleeding, and 2 cases of grade 1 Mucositis. All other AEs presented by maximum grade post-baseline existed at their maximum grade baseline. We summarized and compared the incidence of any-grade AEs by Child-Pugh type (A vs. B vs. C) using a Fisher’s exact test for both max-grade (post-baseline) and baseline adjusted AEs. We did not find a statistically significant difference between overall AE incidence or incidence of any specific AE between Child-Pugh types for either max-grade or baseline adjusted AE incidence rates (at a 0.05 significance level) (Tab. 8).

Overall in the combined data (summarized in Tab. 7), we find the overall incidence of any grade AEs is 78% (similar to that of the Sorafenib trials with overall incidence of any-grade AEs around 80%), although only 17% are grade 3 or 4 AEs. Evaluating baseline adjusted AEs, incidence of any-grade baseline adjusted AEs is 27%, meaning that only 27% of patients present AEs at grades higher than those already experienced at baseline. Among them, only 8% (5 patients) experienced grade 3 or 4 baseline adjusted AEs.

## Discussion

The real-world data presented in this report confirms the TheraBionic device therapeutic activity identified in the previously published phase I/II study. Two (11.1%) of the 18 real-world patients had a PR, which is almost identical to phase I/II study where four (9.8%) of 41 patients had a PR. As a comparison there were only 2/299 (0.67%) and 5/150 (3.33%) PR in the SHARP and Asian-Pacific studies, respectively. The median OS of the six (10.2%) Child-Pugh A and B patients with a PR while receiving treatment with the TheraBionic device was 43.5 months. Five (15.6%) of the 32 Child-Pugh A patients with a PR had a median OS of 52.3 months. Hence, a TheraBionic-induced PR appears to be a predictor of long-term survival. HCC-specific frequencies exhibit pronounced inhibitory effects on cancer stem cells, which may explain the exceptionally long therapeutic response observed in these patients [10]. Of note, one PR occurred in a patient with Child-Pugh B9 disease indicating that severely impaired liver disease does not alter the Thera Bionic device antitumor activity in such patients.

We then assessed whether treatment with the TheraBionic device confers a survival advantage to Child Pugh A and Child Pugh B patients, respectively. We found that the median OS of Child-Pugh A patients is significantly longer than the median placebo OS of matched studies. The median OS of the TheraBionic phase I/II Child-Pugh A patients was 4.64 (64.8%) months longer than the weighted average of the SHARP and Asian-Pacific placebo arms. The median OS of all Child-Pugh A patients who received treatment with the TheraBionic device was 2.62 (33.9%) months longer than the 7.74 months weighted average of median OS from eleven first line and second line studies. When the analysis was restricted to Child-Pugh A patients who did not receive any additional anticancer therapy after initiating treatment with the TheraBionic device, the median OS was 11.2 months indicating that additional anti-cancer therapies did not have a measurable contribution to the survival benefit conferred by the TheraBionic device. Sorafenib is the only TKI recommended by the NCCN guidelines for the treatment of Child-Pugh B disease. Treatment of Child-Pugh B patients with the TheraBionic device as first line therapy resulted in a survival benefit similar to that of Sorafenib in the same group of patients, a large fraction of patients with advanced HCC with limited therapeutic options.

Having shown a statistically significant survival benefit of a magnitude comparable to that of Sorafenib in both Child-Pugh A and Child-Pugh B patients, we sought to assess patient reported symptoms using the same scale as that used in the SHARP and Asian-Pacific Sorafenib studies. None of the patients discontinued treatment with the TheraBionic device even though 27 (45.7%) had either Child-Pugh B or C disease, a subgroup of patients with poorer tolerance to currently approved systemic therapies. After baseline adjustment, the only notable TheraBionic device-related AEs were transient grade 1 mucositis and fatigue, which did not lead to treatment discontinuation. The discrepancy between clinician-rated CTCAE grades and patient-rated adverse events has been well documented. Based on prior research, clinician-rated CTCAE pain grades were 14% lower compared to patient ratings. Therefore, it is possible that we may have detected more robust improvements in disease-related symptoms using patient-reported outcomes measures [23].

In summary, this report provides evidence of a probable survival benefit in patients with Child-Pugh A and B advanced HCC using the TheraBionic device, which is not associated with any adverse event resulting in treatment discontinuation, even in patients with severely impaired liver function. This treatment approach is emerging as a novel therapeutic option for patients who have failed or are intolerant to currently approved therapies and for those patients with severely impaired liver function precluding the use of most currently approved therapies. Treatment with the TheraBionic device is a low risk targeted systemic therapy as it is associated with the delivery of radiofrequency electromagnetic fields at levels substantially lower than those delivered by cellphones [10]. The observed lack of significant adverse events among the very long-term users of the device, patients who experienced a PR, is further evidence of its low risk and associated clinical benefit. While randomized studies represent the golden standard for clinical trials, real-world evidence and/or single arm studies have been compared with historical controls for safety and efficacy assessment of oncology drugs and devices. Specifically, five oncology drugs received FDA approval between 2017 and 2019 based on the use of real-world evidence [24]. The use of such data is exploratory and is reviewed only in order to further characterize the risk:benefit profile of a new treatment modality in the context of the natural history of the disease and treatment outcomes with currently approved therapies. The results presented in this report assess the risk:benefit of the TheraBionic device compared with the natural history of the disease and treatment with Sorafenib, the first TKI approved for advanced HCC, and the only one currently recommended for use in patients with Child-Pugh B7 disease by the NCCN guidelines. This report provides confirmatory evidence of the TheraBionic device safety as well as evidence of probable efficacy comparable to Sorafenib in advanced HCC. This novel therapeutic approach fulfills an unmet need as patients who are eligible or choose TheraBionic are generally ineligible for alternative treatments or do not wish to receive them and are thus not foregoing effective alternatives. This is particularly relevant for patients with severely impaired liver function, an understudied group of patients. The data presented in this report provides support for the use of the TheraBionic device in patients with advanced HCC as a last line of therapy that is both safe and has anticancer activity.

This study has several limitations, mainly the small sample size and selection bias inherent in the use of historical control data. These limitations are in the process of being addressed. In 2020, two separate investigational device exemptions (IDE) were granted by the FDA for further study of the TheraBionic device in advanced HCC. One single arm, single center study is currently assessing the safety and effectiveness of the TheraBionic device in combination with Regorafenib as second line treatment for patients with Child-Pugh A advanced HCC (ClinicalTrials.gov Identifier: NCT04327700). The rationale for this combination is twofold: (1) the fact that TKIs and TheraBionic have complementary antitumor effects as they target different pathways in HCC [10], and (2) two patients who received compassionate use TheraBionic together with a TKI experienced near complete and long-term responses, one of which was recently reported [10]. The FDA granted another IDE for a multicenter, double-blind, randomized study comparing TheraBionic with placebo TheraBionic for the treatment of patients with Child-Pugh A or Child-Pugh B advanced HCC who have failed or are intolerant to at least two lines of systemic therapies (ClinicalTrials.gov Identifier: NCT04797884). This study will assess the safety and effectiveness of the TheraBionic device as a third line therapy in advanced HCC.

## Figures and Tables

**Table 1.** Baseline characteristics of real-world patients. Demographics of 18 real-world patients from the TheraBionic registry. All patients had a diagnosis of advanced HCC and received treatment with HCC-specific frequencies.

Age	Gender	Race	Previous treatment	BCLC stage	Extra hepatic mets	Child-Pugh	*α*-Fetoprotein ≥ 400 ng/mL	ECOG PS	POD prior to treatment

58	M	Caucasian	Yes	C	Yes	A5	Yes	1	Yes
79	M	Caucasian	Yes	C	Yes	A6	Yes	1	Yes
86	M	Caucasian	No	C	No	A5	No	2	No
68	F	Caucasian	Yes	C	Yes	A6	Yes	2	Yes
36	M	Caucasian	Yes	C	Yes	B8	No	2	Yes
84	M	Caucasian	Yes	C	No	A6	No	3	Yes
74	F	Caucasian	Yes	C	Yes	A6	Yes	1	No
79	M	Caucasian	Yes	C	Yes	B9	No	2	Yes
63	M	Arab / Saudi Arabia	Yes	C	Yes	C10	Yes	3	Yes
46	M	Black	Yes	C	Yes	B9	Yes	1	Yes
76	M	Caucasian	Yes	C	Yes	B7	Yes	3	Yes
70	F	Caucasian	Yes	C	No	A6	No	1	Yes
46	M	Caucasian	Yes	C	Yes	A5	No	1	Yes
63	M	Caucasian	Yes	C	No	A5	Yes	2	Yes
74	M	Caucasian	Yes	C	Yes	A6	Yes	2	Yes
75	M	Caucasian	Yes	C	Yes	A6	No	2	Yes
58	M	Caucasian	Yes	C	Yes	A6	No	2	Yes
72	M	Hispanic	Yes	D	No	C11	Yes	2	Yes

**Table 2.** Treatment received by real-world patients prior to treatment with the TheraBionic device. The Table reports the number of patients who received systemic and locoregional treatments prior to treatment with the TheraBionic device.

Prior treatments	Total

Sorafenib	13
Chemoembolization	9
Hepatectomy	2
Nivolumab	2
Radiofrequency ablation	2
Sunitinib	1
SIR-spheres	1
Lenvatinib	1
Stereotactic Body Radiation Therapy	1
Regorafenib	1
Atezolizumab plus Bevacizumab	1
Ytrium-90 radioembolization	1
Cryoablation	1
Pancreatectomy	1
Splenectomy	1
No prior treatment	1

**Table 3.** Adverse Events Summary – Current Study + Phase I/II (*n* = 59). Adverse events reported by NCI CTCAE grade in all patients receiving treatment with the TheraBionic device.

Incidence of adverse events (*n* = 59)												
	Max grade (post-baseline)						Baseline adjusted*					
	Grade 1	Grade 2	Grade 3	Grade 4	Any Grade	Grade 3 or 4	Grade 1	Grade 2	Grade 3	Grade 4	Any Grade	Grade 3 or 4

Overall	32 (54%)	28 (47%)	10 (17%)	1 (2%)	46 (78%)	10 (17%)	10 (17%)	4 (7%)	5 (8%)	1 (2%)	16 (27%)	5 (8%)
Adverse events												
Abdominal pain	3 (5%)	2 (3%)	–	–	5 (8%)	–	–	–	–	–	–	–
Anal hemorrhage	2 (3%)	1 (2%)	–	–	2 (3%)	–	–	–	–	–	–	–
Anemia	1 (2%)	–	–	–	1 (2%)	–	–	–	–	–	–	–
Anorexia	10 (17%)	1 (2%)	1 (2%)	–	10 (17%)	1 (2%)	1 (2%)	–	–	–	1 (2%)	–
Ascites	4 (7%)	9 (15%)	1 (2%)	–	14 (24%)	1 (2%)	–	–	1 (2%)	–	1 (2%)	1 (2%)
Blood in feces	1 (2%)	–	–	–	1 (2%)	–	1 (2%)	–	–	–	1 (2%)	–
Constipation	1 (2%)	–	–	–	1 (2%)	–	–	–	–	–	–	–
Cough	4 (7%)	1 (2%)	–	–	4 (7%)	–	–	–	–	–	–	–
Cramp	1 (2%)	–	–	–	1 (2%)	–	–	–	–	–	–	–
Dehydration	–	–	1 (2%)	–	1 (2%)	1 (2%)	–	–	1 (2%)	–	1 (2%)	1 (2%)
Diarrhea	2 (3%)	2 (3%)	–	–	4 (7%)	–	–	1 (2%)	–	–	1 (2%)	–
Dyspnea	5 (8%)	8 (14%)	–	–	11 (19%)	–	–	1 (2%)	–	–	1 (2%)	–
Edema	2 (3%)	2 (3%)	1 (2%)	–	5 (8%)	1 (2%)	–	–	–	–	–	–
Encephalopathy	2 (3%)	3 (5%)	–	–	5 (8%)	–	–	–	–	–	–	–
Epistaxis	1 (2%)	–	–	–	1 (2%)	–	–	–	–	–	–	–
Fatigue	15 (25%)	7 (12%)	1 (2%)	1 (2%)	21 (36%)	2 (3%)	1 (2%)	–	–	1 (2%)	2 (3%)	1 (2%)
Fever	–	–	1 (2%)	–	1 (2%)	1 (2%)	–	–	1 (2%)	–	1 (2%)	1 (2%)
GI Bleeding	1 (2%)	3 (5%)	–	–	3 (5%)	–	1 (2%)	–	–	–	1 (2%)	–
Hand foot syndrome	1 (2%)	–	–	–	1 (2%)	–	–	–	–	–	–	–
Headache	2 (3%)	–	–	–	2 (3%)	–	1 (2%)	–	–	–	1 (2%)	–
Hepatic Decompensation	1 (2%)	–	–	–	1 (2%)	–	–	–	–	–	–	–
Insomnia	1 (2%)	–	–	–	1 (2%)	–	–	–	–	–	–	–
Jaundice	1 (2%)	1 (2%)	1 (2%)	–	3 (5%)	1 (2%)	–	–	–	–	–	–
Mucositis	4 (7%)	–	–	–	4 (7%)	–	3 (5%)	–	–	–	3 (5%)	–
Myalgia	1 (2%)	–	–	–	1 (2%)	–	1 (2%)	–	–	–	1 (2%)	–
Nausea	2 (3%)	1 (2%)	–	–	3 (5%)	–	–	–	–	–	–	–
Pain	6 (10%)	9 (15%)	6 (10%)	–	18 (31%)	6 (10%)	–	2 (3%)	1 (2%)	–	3 (5%)	1 (2%)
Pruritus	–	2 (3%)	–	–	2 (3%)	–	–	–	–	–	–	–
Somnolence	1 (2%)	–	–	–	1 (2%)	–	1 (2%)	–	–	–	1 (2%)	–
Urinary Tract Infection	1 (2%)	–	–	–	1 (2%)	–	–	–	–	–	–	–
Vertigo	2 (3%)	–	–	–	2 (3%)	–	1 (2%)	–	–	–	1 (2%)	–
Vomiting	1 (2%)	–	–	–	1 (2%)	–	–	–	–	–	–	–
Weakness	11 (19%)	4 (7%)	1 (2%)	–	14 (24%)	1 (2%)	1 (2%)	–	1 (2%)	–	2 (3%)	1 (2%)

* Baseline Adjusted AE grades calculated based on method described by Basch et al. [16].

**Table 4.** Adverse Events Summary – Current Study real-world patients (*n* = 18). Adverse Events reported by NCI CTCAE grade in real-world patients receiving treatment with the TheraBionic device.

Incidence of adverse events (*n* = 18)												
	Max grade (post-baseline)						Baseline adjusted*					
	Grade 1	Grade 2	Grade 3	Grade 4	Any Grade	Grade 3 or 4	Grade 1	Grade 2	Grade 3	Grade 4	Any grade	Grade 3 or 4

Overall	16 (89%)	11 (61%)	1 (6%)	–	18 (100%)	1 (6%)	4 (22%)	–	–	–	4 (22%)	–
Adverse events												
Abdominal pain	1 (6%)	–	–	–	1 (6%)	–	–	–	–	–	–	–
Anal Hemorrhage	2 (11%)	1 (6%)	–	–	2 (11%)	–	–	–	–	–	–	–
Anorexia	9 (50%)	1 (6%)	1 (6%)	–	9 (50%)	1 (6%)	1 (6%)	–	–	–	1 (6%)	–
Ascites	3 (17%)	3 (17%)	–	–	6 (33%)	–	–	–	–	–	–	–
Constipation	1 (6%)	–	–	–	1 (6%)	–	–	–	–	–	–	–
Cough	4 (22%)	1 (6%)	–	–	4 (22%)	–	–	–	–	–	–	–
Diarrhea	2 (11%)	–	–	–	2 (11%)	–	–	–	–	–	–	–
Dyspnea	4 (22%)	5 (28%)	–	–	7 (39%)	–	–	–	–	–	–	–
Edema	1 (6%)	–	–	–	1 (6%)	–	–	–	–	–	–	–
Encephalopathy	1 (6%)	1 (6%)	–	–	2 (11%)	–	–	–	–	–	–	–
Fatigue	10 (56%)	4 (22%)	–	–	12 (67%)	–	–	–	–	–	–	–
GI Bleeding	1 (6%)	1 (6%)	–	–	1 (6%)	–	1 (6%)	–	–	–	1 (6%)	–
Hand foot syndrome	1 (6%)	–	–	–	1 (6%)	–	–	–	–	–	–	–
Jaundice	1 (6%)	1 (6%)	–	–	2 (11%)	–	–	–	–	–	–	–
Mucositis	3 (17%)	–	–	–	3 (17%)	–	2 (11%)	–	–	–	2 (11%)	–
Nausea	2 (11%)	–	–	–	2 (11%)	–	–	–	–	–	–	–
Pain	4 (22%)	2 (11%)	–	–	5 (28%)	–	–	–	–	–	–	–
Pruritus	–	1 (6%)	–	–	1 (6%)	–	–	–	–	–	–	–
Vomiting	1 (6%)	–	–	–	1 (6%)	–	–	–	–	–	–	–
Weakness	10 (56%)	4 (22%)	–	–	12 (67%)	–	–	–	–	–	–	–

* Baseline Adjusted AE grades calculated based on method described by Basch et al. [16].

**Table 5.** Adverse Events Summary – Phase I/II Study (*n* = 41). Adverse Events reported by NCI CTCAE grade in patients receiving treatment with the TheraBionic device.

Incidence of Adverse Events (*n* = 41)												
	Max Grade (Post-Baseline)						Baseline Adjusted*					
	Grade 1	Grade 2	Grade 3	Grade 4	Any Grade	Grade 3 or 4	Grade 1	Grade 2	Grade 3	Grade 4	Any Grade	Grade 3 or 4

Overall	16 (39%)	17 (41%)	9 (22%)	1 (2%)	28 (68%)	9 (22%)	6 (15%)	4 (10%)	5 (12%)	1 (2%)	12 (29%)	5 (12%)
Adverse Events												
Abdominal Pain	2 (5%)	2 (5%)	–	–	4 (10%)	–	–	–	–	–	–	–
Anemia	1 (2%)	–	–	–	1 (2%)	–	–	–	–	–	–	–
Anorexia	1 (2%)	–	–	–	1 (2%)	–	–	–	–	–	–	–
Ascites	1 (2%)	6 (15%)	1 (2%)	–	8 (20%)	1 (2%)	–	–	1 (2%)	–	1 (2%)	1 (2%)
Blood in feces	1 (2%)	–	–	–	1 (2%)	–	1 (2%)	–	–	–	1 (2%)	–
Cramp	1 (2%)	–	–	–	1 (2%)	–	–	–	–	–	–	–
Dehydration	–	–	1 (2%)	–	1 (2%)	1 (2%)	–	–	1 (2%)	–	1 (2%)	1 (2%)
Diarrhea	–	2 (5%)	–	–	2 (5%)	–	–	1 (2%)	–	–	1 (2%)	–
Dyspnea	1 (2%)	3 (7%)	–	–	4 (10%)	–	–	1 (2%)	–	–	1 (2%)	–
Edema	1 (2%)	2 (5%)	1 (2%)	–	4 (10%)	1 (2%)	–	–	–	–	–	–
Encephalopathy	1 (2%)	2 (5%)	–	–	3 (7%)	–	–	–	–	–	–	–
Epistaxis	1 (2%)	–	–	–	1 (2%)	–	–	–	–	–	–	–
Fatigue	5 (12%)	3 (7%)	1 (2%)	1 (2%)	9 (22%)	2 (5%)	1 (2%)	–	–	1 (2%)	2 (5%)	1 (2%)
Fever	–	–	1 (2%)	–	1 (2%)	1 (2%)	–	–	1 (2%)	–	1 (2%)	1 (2%)
GI Bleeding	–	2 (5%)	–	–	2 (5%)	–	–	–	–	–	–	–
Headache	2 (5%)	–	–	–	2 (5%)	–	1 (2%)	–	–	–	1 (2%)	–
Hepatic Decompensation	1 (2%)	–	–	–	1 (2%)	–	–	–	–	–	–	–
Insomnia	1 (2%)	–	–	–	1 (2%)	–	–	–	–	–	–	–
Jaundice	–	–	1 (2%)	–	1 (2%)	1 (2%)	–	–	–	–	–	–
Mucositis	1 (2%)	–	–	–	1 (2%)	–	1 (2%)	–	–	–	1 (2%)	–
Myalgia	1 (2%)	–	–	–	1 (2%)	–	1 (2%)	–	–	–	1 (2%)	–
Nausea	–	1 (2%)	–	–	1 (2%)	–	–	–	–	–	–	–
Pain	2 (5%)	7 (17%)	6 (15%)	–	13 (32%)	6 (15%)	–	2 (5%)	1 (2%)	–	3 (7%)	1 (2%)
Pruritus	–	1 (2%)	–	–	1 (2%)	–	–	–	–	–	–	–
Somnolence	1 (2%)	–	–	–	1 (2%)	–	1 (2%)	–	–	–	1 (2%)	–
Urinary Tract Infection	1 (2%)	–	–	–	1 (2%)	–	–	–	–	–	–	–
Vertigo	2 (5%)	–	–	–	2 (5%)	–	1 (2%)	–	–	–	1 (2%)	–
Weakness	1 (2%)	–	1 (2%)	–	2 (5%)	1 (2%)	1 (2%)	–	1 (2%)	–	2 (5%)	1 (2%)

* Baseline Adjusted AE grades calculated based on method described by Basch et al. [16].

**Table 6.** Adverse Events Summary – Child-Pugh A Patients Only (*n* = 32). Adverse Events reported by NCI CTCAE grade in Child-Pugh A patients receiving treatment with the TheraBionic device.

Incidence of adverse events (*n* = 32)												
	Max grade (post-baseline)						Baseline adjusted*					
	Grade 1	Grade 2	Grade 3	Grade 4	Any Grade	Grade 3 or 4	Grade 1	Grade 2	Grade 3	Grade 4	Any Grade	Grade 3 or 4

Overall	20 (62%)	10 (31%)	5 (16%)	1 (3%)	24 (75%)	5 (16%)	7 (22%)	1 (3%)	2 (6%)	1 (3%)	10 (31%)	2 (6%)
Adverse events												
Abdominal pain	1 (3%)	–	–	–	1 (3%)	–	–	–	–	–	–	–
Anal hemorrhage	2 (6%)	1 (3%)	–	–	2 (6%)	–	–	–	–	–	–	–
Anemia	1 (3%)	–	–	–	1 (3%)	–	–	–	–	–	–	–
Anorexia	7 (22%)	1 (3%)	1 (3%)	–	7 (22%)	1 (3%)	1 (3%)	–	–	–	1 (3%)	–
Ascites	2 (6%)	1 (3%)	1 (3%)	–	4 (12%)	1 (3%)	–	–	1 (3%)	–	1 (3%)	1 (3%)
Blood in feces	1 (3%)	–	–	–	1 (3%)	–	1 (3%)	–	–	–	1 (3%)	–
Constipation	1 (3%)	–	–	–	1 (3%)	–	–	–	–	–	–	–
Cough	3 (9%)	1 (3%)	–	–	3 (9%)	–	–	–	–	–	–	–
Diarrhea	2 (6%)	1 (3%)	–	–	3 (9%)	–	–	1 (3%)	–	–	1 (3%)	–
Dyspnea	4 (12%)	3 (9%)	–	–	6 (19%)	–	–	–	–	–	–	–
Edema	1 (3%)	–	–	–	1 (3%)	–	–	–	–	–	–	–
Encephalopathy	1 (3%)	1 (3%)	–	–	2 (6%)	–	–	–	–	–	–	–
Epistaxis	1 (3%)	–	–	–	1 (3%)	–	–	–	–	–	–	–
Fatigue	10 (31%)	2 (6%)	–	1 (3%)	11 (34%)	1 (3%)	1 (3%)	–	–	1 (3%)	2 (6%)	1 (3%)
Hand foot syndrome	1 (3%)	–	–	–	1 (3%)	–	–	–	–	–	–	–
Headache	1 (3%)	–	–	–	1 (3%)	–	–	–	–	–	–	–
Hepatic Decompensation	1 (3%)	–	–	–	1 (3%)	–	–	–	–	–	–	–
Jaundice	–	1 (3%)	–	–	1 (3%)	–	–	–	–	–	–	–
Mucositis	3 (9%)	–	–	–	3 (9%)	–	2 (6%)	–	–	–	2 (6%)	–
Nausea	1 (3%)	–	–	–	1 (3%)	–	–	–	–	–	–	–
Pain	4 (12%)	1 (3%)	3 (9%)	–	8 (25%)	3 (9%)	–	–	1 (3%)	–	1 (3%)	1 (3%)
Pruritus	–	1 (3%)	–	–	1 (3%)	–	–	–	–	–	–	–
Urinary Tract Infection	1 (3%)	–	–	–	1 (3%)	–	–	–	–	–	–	–
Vertigo	1 (3%)	–	–	–	1 (3%)	–	1 (3%)	–	–	–	1 (3%)	–
Vomiting	1 (3%)	–	–	–	1 (3%)	–	–	–	–	–	–	–
Weakness	8 (25%)	2 (6%)	–	–	8 (25%)	–	1 (3%)	–	–	–	1 (3%)	–

* Baseline Adjusted AE grades calculated based on method described by Basch et al. [16].

**Table 7.** Incidence of any grade adverse events by Child-Pugh Type. Adverse Events reported by Child-Pugh type in patients receiving treatment with the TheraBionic device. Fisher’s exact test used for statistical comparison of reported incidence of AEs. Statistical significance in adverse event incidence reported at a 0.05 significance level.

	Max-grade (post-baseline)				Baseline adjusted*			
	Child-Pugh Type			Fisher’s Exact Test	Child-Pugh Type			Fisher’s Exact Test
	Type A	Type B	Type C	*P*-value	Type A	Type B	Type C	*P*-value

Overall	24 (75%)	19 (76%)	2 (100%)	>0.99	10 (31%)	6 (24%)	0 (0%)	0.877
Adverse Events								
Abdominal pain	1 (3%)	4 (16%)	0 (0%)	0.295	0 (0%)	0 (0%)	0 (0%)	>0.99
Anal hemorrhage	2 (6%)	0 (0%)	0 (0%)	0.532	0 (0%)	0 (0%)	0 (0%)	>0.99
Anemia	1 (3%)	0 (0%)	0 (0%)	>0.99	0 (0%)	0 (0%)	0 (0%)	>0.99
Anorexia	7 (22%)	3 (12%)	0 (0%)	0.647	1 (3%)	0 (0%)	0 (0%)	>0.99
Ascites	4 (12%)	8 (32%)	2 (100%)	0.012	1 (3%)	0 (0%)	0 (0%)	>0.99
Asthenia	0 (0%)	0 (0%)	0 (0%)	>0.99	0 (0%)	0 (0%)	0 (0%)	>0.99
Blood in feces	1 (3%)	0 (0%)	0 (0%)	>0.99	1 (3%)	0 (0%)	0 (0%)	>0.99
Constipation	1 (3%)	0 (0%)	0 (0%)	>0.99	0 (0%)	0 (0%)	0 (0%)	>0.99
Cough	3 (9%)	1 (4%)	0 (0%)	0.673	0 (0%)	0 (0%)	0 (0%)	>0.99
Cramp	0 (0%)	1 (4%)	0 (0%)	0.458	0 (0%)	0 (0%)	0 (0%)	>0.99
Dehydration	0 (0%)	1 (4%)	0 (0%)	0.458	0 (0%)	1 (4%)	0 (0%)	0.458
Diarrhea	3 (9%)	1 (4%)	0 (0%)	0.673	1 (3%)	0 (0%)	0 (0%)	>0.99
Dyspnea	6 (19%)	4 (16%)	1 (50%)	0.462	0 (0%)	1 (4%)	0 (0%)	0.458
Edema	1 (3%)	4 (16%)	0 (0%)	0.295	0 (0%)	0 (0%)	0 (0%)	>0.99
Encephalopathy	2 (6%)	2 (8%)	1 (50%)	0.214	0 (0%)	0 (0%)	0 (0%)	>0.99
Epistaxis	1 (3%)	0 (0%)	0 (0%)	>0.99	0 (0%)	0 (0%)	0 (0%)	>0.99
Fatigue	11 (34%)	8 (32%)	2 (100%)	0.209	2 (6%)	0 (0%)	0 (0%)	0.532
Fever	0 (0%)	1 (4%)	0 (0%)	0.458	0 (0%)	1 (4%)	0 (0%)	0.458
GI bleeding	0 (0%)	3 (12%)	0 (0%)	0.171	0 (0%)	1 (4%)	0 (0%)	0.458
Hand foot syndrome	1 (3%)	0 (0%)	0 (0%)	>0.99	0 (0%)	0 (0%)	0 (0%)	>0.99
Headache	1 (3%)	1 (4%)	0 (0%)	>0.99	0 (0%)	1 (4%)	0 (0%)	0.458
Hepatic decompensation	1 (3%)	0 (0%)	0 (0%)	>0.99	0 (0%)	0 (0%)	0 (0%)	>0.99
Hyperoxia	0 (0%)	0 (0%)	0 (0%)	>0.99	0 (0%)	0 (0%)	0 (0%)	>0.99
Insomnia	0 (0%)	1 (4%)	0 (0%)	0.458	0 (0%)	0 (0%)	0 (0%)	>0.99
Jaundice	1 (3%)	1 (4%)	1 (50%)	0.1	0 (0%)	0 (0%)	0 (0%)	>0.99
Mucositis	3 (9%)	1 (4%)	0 (0%)	0.673	2 (6%)	1 (4%)	0 (0%)	>0.99
Myalgia	0 (0%)	1 (4%)	0 (0%)	0.458	0 (0%)	1 (4%)	0 (0%)	0.458
Nausea	1 (3%)	2 (8%)	0 (0%)	0.619	0 (0%)	0 (0%)	0 (0%)	>0.99
Pain	8 (25%)	8 (32%)	1 (50%)	0.517	1 (3%)	2 (8%)	0 (0%)	0.619
Pruritus	1 (3%)	1 (4%)	0 (0%)	>0.99	0 (0%)	0 (0%)	0 (0%)	>0.99
Somnolence	0 (0%)	1 (4%)	0 (0%)	0.458	0 (0%)	1 (4%)	0 (0%)	0.458
Urinary tract infection	1 (3%)	0 (0%)	0 (0%)	>0.99	0 (0%)	0 (0%)	0 (0%)	>0.99
Vertigo	1 (3%)	1 (4%)	0 (0%)	>0.99	1 (3%)	0 (0%)	0 (0%)	>0.99
Vomiting	1 (3%)	0 (0%)	0 (0%)	>0.99	0 (0%)	0 (0%)	0 (0%)	>0.99
Weakness	8 (25%)	4 (16%)	2 (100%)	0.054	1 (3%)	1 (4%)	0 (0%)	>0.99

* Baseline Adjusted AE grades calculated based on method described by Basch et al. [16].

**Table 8.** Adverse Events Summary – Comparison to Sorafenib Trials. Adverse Events reported by phase I/II study patients receiving treatment with the TheraBionic device or Sorafenib (SHARP/Asian-Pacific). Statistical significance in adverse event incidence reported at a 0.05 significance level.

Adverse event	Incidence of any grade AEs				
	TheraBionic (*N* = 41)	SHARPstudy [17] (*N* = 297)		Asian-Pacific study [18] (*N* = 149)	
	*N* (%)	*N* (%)	*P*-value	*N* (%)	*P*-value

Abdominal pain	4 (10%)	24 (8%)	0.761	–	–
Anorexia	1 (2%)	41 (14%)	0.041	19 (13%)	0.081
Diarrhea	2 (5%)	116 (39%)	<0.001	38 (26%)	0.004
Fatigue	9 (22%)	65 (22%)	1	30 (20%)	0.828
Hand foot syndrome	0 (0%)	62 (21%)	<0.001	67 (45%)	<0.001
Nausea	1 (2%)	33 (11%)	0.099	17 (11%)	0.129

Note: *P*-values based on Fisher’s Exact Test comparing TheraBionic and each Sorafenib Trial.

## Nomenclature

AE: Adverse events
AFP: Alpha-fetoprotein
AM: Amplitude-modulated
BCLC: Barcelona-Clinic Liver Cancer
CACNA1H: Voltage-gated calcium channel alpha subunit 3.2
Cav3.2: Voltage-gated calcium channel alpha subunit 3.2
CE: Conformité européene (European conformity)
CI: Confidence interval
CRF: Case report form
CTCAE: Common terminology criteria for adverse events
ECOG: Eastern cooperative oncology group
EEC: European Economic Community
EMF: Electromagnetic fields
FDA: Food and drug administration
GI: Gastrointestinal
HCC: Hepatocellular carcinoma
IDE: Investigational device exemption
IP3/DAG: Inositol-1,4,5-triphosphate diacylglycerol
ISO: International Organization for Standardization
MDD: Medical Device Directive
MEDDEV: MEDical DEVice Documents
mL: milliliter
mRECIST: Modified response evaluation criteria in solid tumors
ng: nanogram
NCI: National Cancer Institute
NCCN: National Comprehensive Cancer Network
OS: Overall survival
POD: Progression of disease
PRO: Patient-reported outcome
PR: Partial response
PS: Performance status
RECIST: Response evaluation criteria in solid tumors
RECIST 1.1: Response evaluation criteria in solid tumors (version 1.1)
RF: Radiofrequency
SIR: Selective internal radiation
TDRF: TheraBionic device registry form
TKI: Tyrosine Kinase Inhibitor
TTP: Time to progression
VGCC: Voltage gated calcium channel
